# Ultrasound Targeted Microbubble Destruction Stimulates Cellular Endocytosis in Facilitation of Adeno-Associated Virus Delivery

**DOI:** 10.3390/ijms14059737

**Published:** 2013-05-07

**Authors:** Li-Fang Jin, Fan Li, Hui-Ping Wang, Fang Wei, Peng Qin, Lian-Fang Du

**Affiliations:** 1 Department of Ultrasound, Shanghai Jiaotong University Affiliated First People’s Hospital, 100 Haining Road, Shanghai 200080, China; E-Mails: lifang_jin@163.com (L.-F.J.); medicineli@163.com (F.L.); 2 Experimental Research Center, Shanghai Jiaotong University Affiliated First People’s Hospital, 100 Haining Road, Shanghai 200080, China; E-Mails: tywhp9618@msn.com (H.-P.W.); weifang97@gmail.com (F.W.); 3 Department of Instrumentation Science and Engineering, Shanghai Jiaotong University, 800 Dongchuan Road, Shanghai 200240, China; E-Mail: pqin@sjtu.edu.cn

**Keywords:** ultrasound, microbubbles, endocytosis, adeno-associated virus, sonoporation

## Abstract

The generally accepted mechanism for ultrasound targeted microbubble destruction (UTMD) to enhance drug and gene delivery is through sonoporation. However, passive uptake of adeno-associated virus (AAV) into cells following sonoporation does not adequately explain observations of enhanced transduction by UTMD. This study investigated alternative mechanisms of UTMD enhancement in AAV delivery. UTMD significantly enhanced transduction efficiency of AAV in a dose-dependent manner. UTMD stimulated a persistent uptake of AAV into the cytoplasm and nucleus. This phenomenon occurred over several hours, suggesting that some viral particles are endocytosed by cells rather than exclusively passing through pores created by sonoporation. Additionally, UTMD enhanced clathrin expression and accumulation at the plasma membrane suggesting greater clathrin-mediated endocytosis following UTMD. Transmission electron microscopy (TEM) revealed that UTMD stimulated formation of clathrin-coated pits (CPs) and uncoated pits (nCPs). Furthermore, inhibition of clathrin-mediated endocytosis partially blocked the enhancement of AAV uptake following UTMD. The results of this study implicate endocytosis as a mechanism that contributes to UTMD-enhanced AAV delivery.

## 1. Introduction

Ultrasound targeted microbubble destruction (UTMD) has been recognized as an efficient modality for drug and gene delivery [1–3]. Microbubble destruction by ultrasound exposure generates microstreams or microjets that create shear stress on cells and open transient pores in cell membranes [4–6]. This phenomenon is known as sonoporation [6–9] and is thought to facilitate delivery of extracellular material such as nucleic acids, drugs, and viruses by allowing passive movement of material through pores created by UTMD [7,10–12].

UTMD has been shown to enhance gene transduction of adeno-associated virus (AAV) [13,14]. Endocytosis, and especially clathrin-mediated endocytosis, is a critical component of AAV transduction [15], as direct injection of AAV into the cytoplasm results in less transduction than cellular incubation with virus in the extracellular medium [16,17]. Therefore, it would be expected that passive entry into the cytoplasm following sonoporation should result in less efficient transduction and does not adequately explain the enhancement of AAV transduction observed after UTMD.

Previous studies have cast doubt on sonoporation being the exclusive mechanism of UTMD-enhanced cellular uptake. These studies observed pore-like structures in cells that were not treated with ultrasound, as well as cells exposed to ultrasound with or without microbubbles [18]. Furthermore, atomic-force microscopy suggests that these pore-like structures were simply depressions in the membrane rather than actual pores [19]. UTMD has been shown to induce formation of hydrogen peroxide (H_2_O_2_) and increase Ca^2+^ influx [20], which correlated with endocytosis [21,22]. Recently, endocytosis has been shown to be essential for UTMD-mediated delivery of dextran and fluorescent molecular tags [23–25].

In light of the evidence that implicates increased endocytosis following UTMD, this study investigates the relationship between UTMD and clathrin-mediated endocytosis in UTMD-mediated AAV delivery in HeLa cells. Dose-dependent enhanced gene transduction of AAV was observed and AAV particles were detected in the cytoplasm and nucleus. Levels of clathrin-mediated endocytosis following UTMD were measured, and the upregulation of clathrin-mediated endocytosis in AAV-mediated gene transduction following UTMD was demonstrated.

## 2. Results

### 2.1. UTMD Enhances the Gene Transduction of AAV in a Dose-Dependent Manner

**Figure 1:**
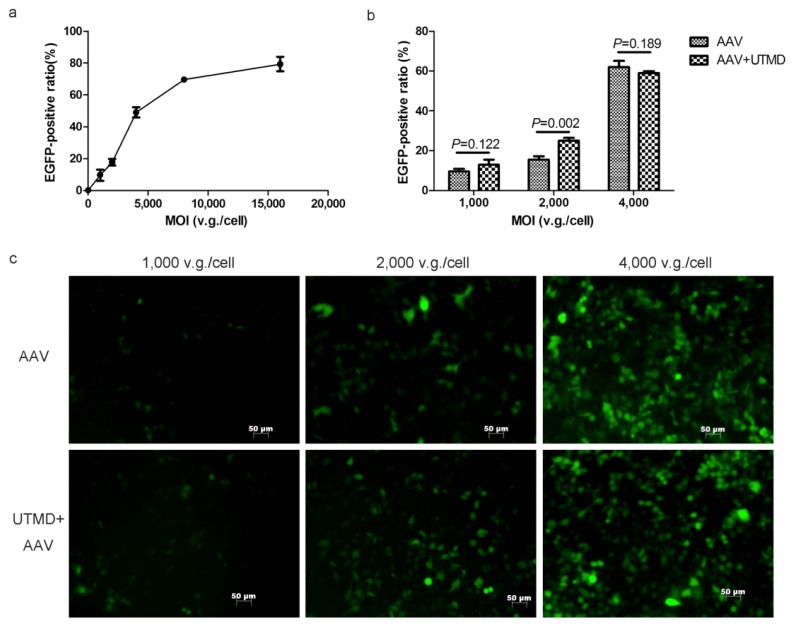
The dose-dependence of UTMD-enhanced AAV transduction of HeLa cells. (**a**) The dose-effect curve of AAV transduction. Ratios of EGFP expression were determined by flow cytometry; (**b**) Transduction efficiency with and without UTMD at various doses of AAV; (**c**) Fluorescence microscopy of EGFP transduction with and without UTMD at various doses of AAV.

Using the dose-effect curve, 2000 vector genome (v.g.)/cell was selected for its position in the middle of the ascending segment of the curve. A lower multiplicity of infection (MOI) of 1000 v.g./cell and higher MOI of 4000 v.g./cell were selected to examine the dose-dependence of UTMD enhancement. The MOIs of 1000 v.g./cell, 2000 v.g./cell, and 4000 v.g./cell represented low, medium, and high doses, respectively (Figure 1a). A significant enhancement was observed when cells were exposed to AAV at the medium dose of 2000 v.g./cell (*p* = 0.002). Transduction efficiency was enhanced from 15.56% ± 1.64% to 24.96% ± 1.42% and the average fold enhancement was 1.60. However, UTMD did not enhance transduction at the low and high doses (Figure 1b,c). The UTMD enhancement of AAV transduction was dose-dependent and most efficient at the medium dose. Therefore, the MOI of 2000 v.g./cell was used in all of the following experiments.

### 2.2. UTMD Facilitates Viral Entry into the Cytoplasm and Nucleus

**Figure 2:**
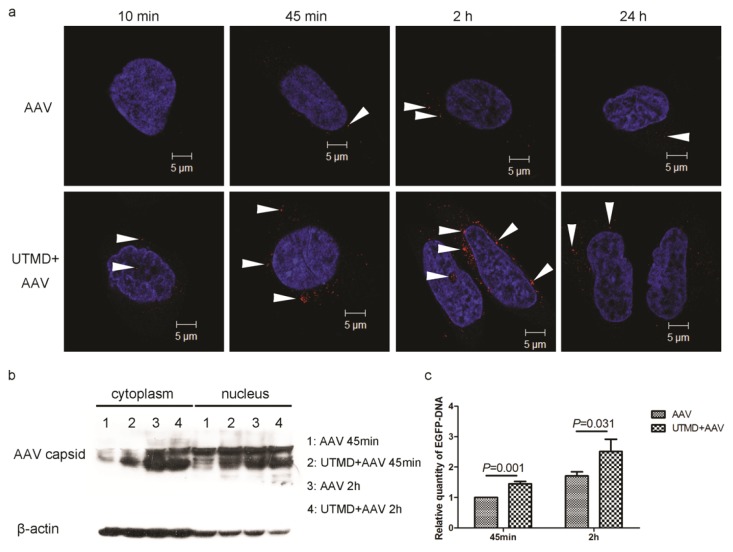
UTMD-mediated enhancement of AAV entry into HeLa cells. (**a**) Time-course of AAV uptake in HeLa cells with or without UTMD. AAV were added at an MOI of 2000 v.g./cell. Confocal immunofluorescence microscopy shows AAV (red, indicated by arrowheads) and nuclei (blue); (**b**) Quantities of AAV2 capsid protein in the cytoplasm or nuclei of AAV-transfected HeLa cells with and without UTMD at 45 min and 2 h post-infection; (**c**) Relative quantities of EGFP DNA following AAV transduction of HeLa cells with and without UTMD at 45 min and 2 h post-infection.

Confocal immunofluorescence microscopy revealed that the number of AAV particles in HeLa cells increased over time and peaked at 2 h post-infection in the AAV group. After UTMD, cell-associated AAV increased at all time points, but most importantly, enhancement peaked between 45 min and 2 h post-treatment. Additionally, viral entry into the nucleus was enhanced by UTMD at 10 min and 2 h post treatment (Figure 2a). This observation demonstrated the enhanced viral uptake following UTMD continued for several hours and did not reach its peak immediately after UTMD.

Because the quantity of AAV capsids is proportional to the number of viral particles, AAV entry can be quantified by measuring the levels of cell-associated AAV capsid protein. In both AAV group and UTMD+AAV group, the relative quantity of AAV capsid protein in the cytoplasm and nucleus increased with AAV incubation time (45 min to 2 h). When incubated for 45 min, AAV uptake into the cytoplasm and nucleus was increased by UTMD treatment. However, UTMD did not significantly enhance AAV uptake during the 2 h incubation (Figure 2b). The greater amount of AAV protein in the cytoplasm and nucleus following UTMD suggests that UTMD-facilitated viral entry into the cytoplasm and nucleus by 45 min post-treatment.

Enhanced green fluorescent protein (EGFP) DNA carried by AAV was quantified by PCR. The quantity of DNA was proportional to the number of viral particles. UTMD significantly enhanced the relative quantity of EGFP DNA taken up by cells at 45 min and 2 h post-treatment by 1.45 ± 0.08 (*p* = 0.001) and 1.46 ± 0.13 (*p* = 0.031) fold, respectively (Figure 2c).

### 2.3. UTMD Stimulates Cellular Endocytosis

**Figure 3:**
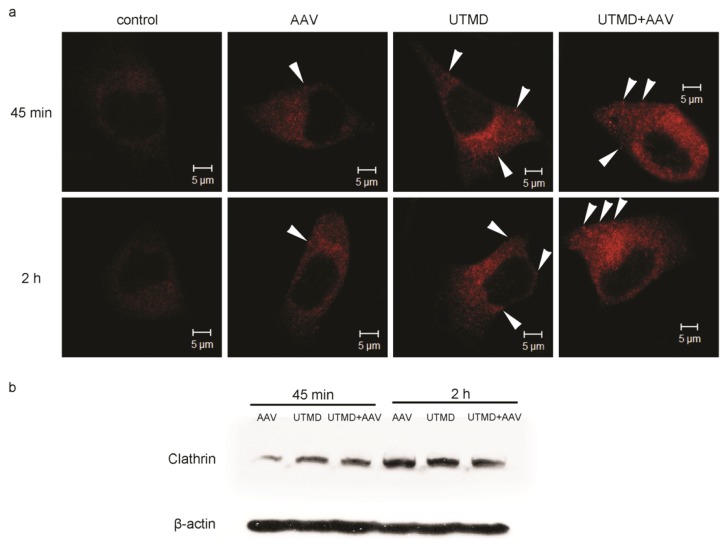
UTMD-mediated enhancement of clathrin expression and accumulation at the plasma membrane. (**a**) Clathrin localization in HeLa cells 45 min and 2 h post-infection with AAV. Clathrin appears red and fluorescent foci are indicated by white arrowheads. Untreated cells (no AAV, no UTMD) served as the control group; (**b**) Clathrin quantification by western blot in HeLa cells treated with AAV, UTMD, or UTMD+AAV at 45 min and 2 h post-treatment.

In both control (untreated) and AAV infected cells, clathrin was distributed uniformly throughout the cytoplasm. In the UTMD treated and UTMD+AAV treated groups, clathrin was observed as cytoplasmic foci near the cell membrane. Additionally, the fluorescence intensity in AAV infected cells was higher than in control cells, but lower than in UTMD-treated and UTMD+AAV-treated cells (Figure 3a). The foci indicate increased accumulation of clathrin in clathrin-coated endocytic vesicles, which were increased following UTMD.

To further investigate the enhancement of endocytosis, clathrin was quantified by western blot. Regardless of which treatment cells received, levels of clathrin at 2 h post-treatment (AAV and/or UTMD) were higher than at 45 min post-treatment. Higher levels indicated that the synthesis of clathrin could be stimulated. In the treatment groups, greater quantities of clathrin were observed in UTMD-treated and UTMD+AAV-treated cells at 45 min post infection compared to cells that were not treated with UTMD. However, there were no differences between UTMD-treated and UTMD-untreated groups at 2 h post infection (Figure 3b). Levels of clathrin in the UTMD+AAV group at 2 h post-treatment had fewer positive imaging features for edge effect.

**Figure 4:**
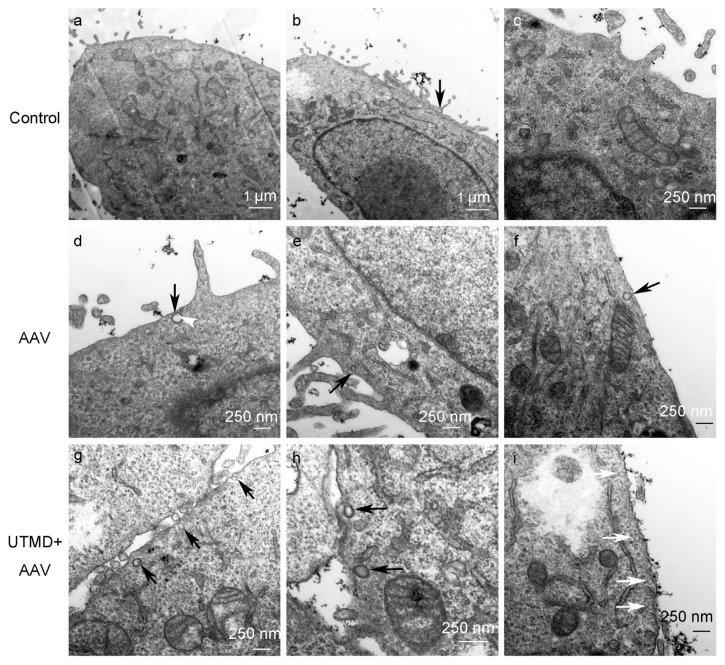
TEM of HeLa cells not treated, treated with AAV or UTMD+AAV at 45 min post-treatment. CPs (coated with thick, coarse cyst walls) are indicated by black arrows and nCPs (with cyst walls that are thin and smooth) are indicated by white arrows. AAV particles are indicated by white arrowheads. Untreated cells served as the control group.

To examine endocytic vesicles directly, cells were imaged by transmission electron microscopy (TEM). In control cells, endocytic vesicles were rarely found near the plasma membrane. In AAV-infected cells, a few clathrin-coated pits (CPs) were observed near the plasma membrane and in UTMD+AAV treated cells, even greater numbers of CPs and uncoated pits (nCPs) were observed (Figure 4).

### 2.4. UTMD-Enhanced Transduction of AAV Partially Depends on Clathrin-Mediated Endocytosis

**Figure 5:**
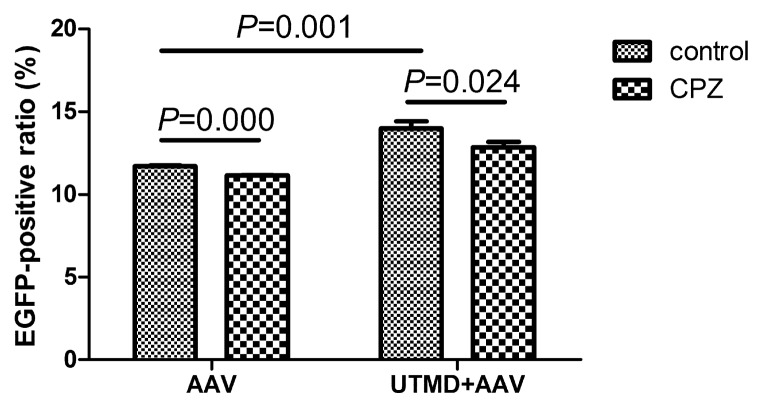
Inhibition of clathrin-mediated endocytosis by CPZ reduces UTMD-enhanced transduction in HeLa cells treated with AAV alone or UTMD+AAV.

Chlorpromazine (CPZ) was used to inhibit clathrin-mediated endocytosis. Treatment with CPZ significantly reduced the levels of AAV transduction in cells that were not treated with UTMD from 11.71% ± 0.05% to 11.14% ± 0.03% (*p* = 0.000). Moreover, CPZ significantly reduced AAV transduction following UTMD treatment from 13.98% ± 0.45% to 12.85% ± 0.33% (*p* = 0.024). The mean reduction in transduction efficiency by CPZ in the UTMD+AAV group was 1.13%, which was greater than 0.57% in the AAV group (Figure 5). Because CPZ inhibits clathrin-mediated endocytosis, the decreased transduction without UTMD suggests viral uptake by cells is partially dependent on clathrin-mediated endocytosis. Greater inhibition of uptake following UTMD after treatment with CPZ indicates UTMD-mediated enhancement of transduction is also partially dependent on clathrin-mediated endocytosis.

## 3. Discussion

This study demonstrated UTMD stimulated endocytosis in HeLa cells increases AAV uptake in a delayed manner, and enhances gene transduction of AAV in a dose-dependent manner. Furthermore, the enhanced AAV uptake was dependent on clathrin-mediated endocytosis suggesting that sonoporation was not the exclusive mechanism of viral entry into cells following UTMD.

The inability for UTMD to further enhance viral uptake at the high dose suggests competitive inhibition and saturation of endocytosis machinery. AAV had been shown to primarily enter cells by receptor-mediated endocytosis using clathrin-coated pits [26–28]. Given the finite number of binding sites and corresponding endocytic vesicles in each cell, competitive inhibition of viral endocytosis occurs at high concentrations of AAV. In contrast, passive movement through pores created by sonoporation does not adequately explain the observed saturation of uptake.

Results from confocal immunofluorescence microscopy, real-time PCR, and western blot (Figure 2) demonstrate that AAV internalization in cells is increased by UTMD and that enhanced internalization continues for several hours after UTMD. We showed that the peak time of enhancement occurred around 45 min after UTMD, which corresponds to the time-course of uptake through endocytosis [26–28]. This evidence suggests that UTMD stimulates endocytosis of AAV. The increase of clathrin accumulation and activity implies that clathrin-mediated endocytosis is activated following UTMD.

No obvious enhancement in AAV entry or activity of clathrin-mediated endocytosis was detected by western blot, a less sensitive technique, at 2 h after UTMD. Therefore, 45 min post-treatment was selected for the experimental time point for TEM. In TEM, the direct relationship between UTMD and endocytosis is confirmed by the increased endocytic vehicles inside the cellular membranes.

Interestingly, TEM revealed that UTMD stimulated formation of both clathrin CPs and nCPs. This evidence indicates that UTMD stimulates clathrin-mediated endocytosis as well as other endocytic pathways. It was reported that AAV enters cells partially through nCPs [28,29]. Therefore, it is possible that UTMD enhances cellular uptake of AAV through both clathrin-mediated endocytosis [15,30], as well as other endocytic pathways to a lesser extent. Further research is needed to confirm the participation of other endocytic pathways in UTMD-mediated enhancement of AAV transduction.

In the current study, the human cervical carcinoma cell line HeLa was selected for two reasons. First, HeLa cells were previously used in many studies of cellular AAV trafficking [27,31–33] that are relevant to our study, so HeLa cells were chosen to compare our results to other cell trafficking studies. Second, HeLa cells are strongly adherent and therefore tolerate UTMD well as cells can shrink dramatically during UTMD. Other cell lines will also be tested in the future.

However, there were some limitations in the current study. First, we only examined clathrin-mediated endocytosis. There are several pathways for endocytosis such as clathrin-meditated endocytosis and non-clathrin-mediated endocytosis [34]. Because clathrin-mediated endocytosis is the most common entry mechanism for AAV trafficking [15] and the focus of the current study was AAV trafficking, clathrin-mediated endocytosis was the most reasonable mechanism to investigate. Other pathways indicated by TEM observations might also contribute to UTMD enhancement in gene transduction of AAV. Second, only one pharmacological agent (CPZ) was used to inhibit the clathrin-mediated endocytosis. CPZ redistributes adaptor complex 2 and clathrin away from the plasma membrane, which makes clathrin unavailable for assembly at the cell surface [35]. Because CPZ perturbs the formation of clathrin-coated vesicles and UTMD is thought to stimulate the formation of these vesicles, CPZ was selected as the inhibitor. Other inhibitors such as phenylarsine oxide and monodansylcadaverine, which act on other steps in clathrin-mediated endocytosis may be worthwhile to investigate as well [36]. Ultimately, we observed only small reductions in gene transduction efficiency. It is possible that clathrin-mediated endocytosis was not responsible for AAV entry exclusively. Other pathways, such as caveolin-mediated endocytosis and microtubule network [28,37,38], are also important for AAV entry. Further research on other pathways should be performed.

Our results indicated that a stimulation of clathrin-mediated endocytosis is partly responsible for UTMD-enhanced AAV delivery rather than the process being entirely dependent on sonoporation. Using UTMD to stimulate endocytosis could be beneficial for AAV transduction in cell types with endocytosis defects [39,40] and could potentially improve strategies for gene delivery.

## 4. Experimental Section

### 4.1. Cell Culture

HeLa cells were maintained in Dulbecco’s Modified Eagle Medium (DMEM, Gibco, New York, NY, USA) at 37 °C in 5% CO_2_. The medium was supplemented with 10% fetal bovine serum (Gibco, New York, NY, USA).

### 4.2. UTMD Protocols

A therapeutic ultrasound machine (PHYSIOSON-Basic, PHYSIOSON Elektro-medizin, Germany) was used to emit ultrasound at the frequency of 1 MHz. The area of the ultrasound probe was 2.5 cm^2^. The ultrasound transducer was placed at the bottom of plates or dishes with coupling medium on the surface of the transducer. The adjustable sonication parameters included ultrasound intensity, exposure time, pulse frequency, and duty cycle.

Microbubbles (SonoVue, Bracco, Milan, Italy) were lipid-shelled ultrasound contrast agents containing sulfur hexafluoride gas (diameter 2.5–6.0 μm) and used at a concentration of ~2 × 10^8^ bubbles/mL. The volumetric ratio of microbubbles to medium dictated the choice of contrast agent dose.

After protocol optimization with several various settings, the following UTMD parameters were used: ultrasound intensity, 1 W/cm^2^; exposure time, 60 s; pulse frequency, 100 Hz; duty cycle, 20%; volumetric ratio of microbubbles:medium, 1:5.

### 4.3. Gene Transduction Efficiency Assays

Viral transduction efficiency assays were used in the dose-dependence experiments and inhibition experiments. HeLa cells were seeded into 24-well plates at a density of 1 × 10^5^ cells/well for 24 h before infection. Cells were infected by adding 150 μL of complete DMEM containing a recombinant AAV type-2 vector encoding the EGFP gene (rAAV2-EGFP, Beijing FivePlus Molecular Medicine Institute, Beijing, China) with or without microbubbles. Cells were treated with ultrasound immediately after adding microbubbles then incubated for 2 h. After 2 h, 350 μL of fresh DMEM was added to the wells, and at 24 h post-treatment, medium containing the virus was replaced with 500 μL of complete DMEM.

A dose-dependent curve of transduction was generated using the following viral doses expressed as a multiplicity of infection (MOI): 0 v.g./cell; 1000 v.g./cell; 2000 v.g./cell; 4000 v.g./cell; 8000 v.g./cell; and 16,000 v.g./cell. MOIs of 1000 v.g./cell, 2000 v.g./cell, and 4000 v.g./cell were selected for dose-dependence experiments. An MOI of 2000 v.g./cell was chosen for the inhibition experiments.

At 48 h post-treatment, EGFP transduction efficiency was evaluated by fluorescence microscopy and flow cytometry. Green fluorescence was detected using an inverted fluorescence microscopy (ZEISS Axiovert S 100, Jena, Germany). The percentage of infected cells was measured by flow cytometry (FACSCalibur, Becton Dickinson, Franklin Lakes, NJ, USA) following trypsinization and centrifugation of cells. Treatment groups were AAV (treatment with rAAV2-EGFP alone, no UTMD) and UTMD+AAV (treatment with rAAV2-EGFP followed by UTMD). Measurements for each group were done in triplicate (1 well per replicate).

### 4.4. Confocal Immunofluorescence Microscopy

HeLa cells were plated in 35-mm dishes with 15 mm sunken glass bottoms at a density of 2 × 10^5^ cells per dish 24 h before infection. For treatment with AAV and UTMD, 150 μL of medium containing virus and/or microbubbles was added to the sunken glass bottoms. At various time points post-treatment, cells were washed 3 times with PBS, fixed with 4% paraformaldehyde for 15 min, permeabilized with 0.5% Triton X-100 for 5 min, and then blocked with 10% BSA for 45 min at room temperature. Cells were then incubated with primary antibody (Mab to AAV intact particles (PROGEN 61055, Heidelberg, Germany, 1:20), clathrin heavy chain polyclonal antibody (Pierce PA5-17347, Rockford, AL, USA, 1:200)) overnight at 4 °C. After 3 washes with PBS, cells were incubated in secondary antibody (Alexa Fluor 594 labeled donkey anti-mouse IgG (Invitrogen, Eugene, OR, USA; 1:200), Alexa Fluor 594 labeled donkey anti-rabbit IgG (Invitrogen, Eugene, OR, USA; 1:200)) for 1 h at 37 °C. DAPI (5 μg/mL, Dojindo, Japan) was used to label nuclei. Cells were maintained in PBS prior to imaging on a laser scanning confocal microscope (Carl Zeiss LSM-510, Jena, Germany). Image processing was performed with Adobe Photoshop.

### 4.5. Real-Time PCR

At 45 min and 2 h post-treatment with AAV and UTMD, 6 wells of cells for each group were trypsinized and harvested from 24-well plates. Cells were lysed and the total DNA was extracted from samples using Universal Genomic DNA Extraction Kit Ver.3.0 (Takara DV811A, Dalian, China) according to the manufacturer’s protocol. The quantity and purity of the isolated DNA were measured by spectrophotometry. Quantitative PCR was performed on a DNA Engine Opticon™ 2 (MJ research, Waltham, QC, USA) using a SYBR^®^ Premix Ex Taq™ kit (Takara DV811A, Dalian, China) using the following primers for EGFP: 5′-AGAAGAA CGGCATCAAGGTG-3′ (forward) and 5′-GAACTCCAGCAGGACCATGT-3′ (reverse). Reaction conditions were as follows: 1 cycle at 95 °C for 30 s; 35 cycles at 95 °C for 5 s, 62 °C for 20 s, and 72 °C for 20 s. Quantification, using the 2^−ΔΔCT^ analytical method, was performed in triplicate with β-actin as the internal standard.

### 4.6. Cell Fractionation

At 45 min and 2 h post-treatment with AAV and UTMD, 24 wells of cells for each group were trypsinized and harvested from 24-well plates. Cytoplasmic and nuclear proteins were extracted from samples using NE-PER^®^ Nuclear and Cytoplasmic Extraction Reagents, respectively (Pierce 78833, Rockford, AL, USA) according to manufacturer protocols.

### 4.7. Western Blotting

The quantity of isolated proteins was measured using a bicinchoninic acid (BCA) assay. To detect target proteins, samples were separated by sodium dodecyl sulfate- polyacrylamide gel electrophoresis (SDS-PAGE) and transferred to nitrocellulose membranes. Membranes were blocked with milk for 2 h at room temperature. After incubation with a primary antibody (Mab to VP 1, VP 2 and VP 3 of AAV (PROGEN 61058, Heidelberg, Germany, 1:100), Clathrin heavy chain polyclonal antibody (Pierce PA5-17347, Rockford, AL, USA, 1:1000)) overnight at 4 °C and three washing steps in Tris-buffered saline supplemented with Tween 20 (TBST), the membrane was incubated for 2 h with a secondary antibody (HRP-linked anti-mouse IgG antibody (CST 7076, 1:1,000), HRP-linked anti-rabbit IgG antibody (CST 7074, 1:1000)). The membrane was washed again and incubated for 1 min with SuperSignal West Pico Chemiluminescent Substrate (Pierce). Membranes were exposed to Biomax Light Film (Kodak) for development. Protein levels were normalized to β-actin levels.

### 4.8. Transmission Electron Microscopy

HeLa cells grown in 24-well plates were infected with AAV and treated with UTMD. At 45 min post-infection, cells were fixed at room temperature in 2% glutaraldehyde for 2 h, followed by 2 h incubation in 1% OsO_4_ prior to dehydration and embedding. Microscopic images were captured on a transmission electron microscope (Hitachi H-7650, Tokyo, Japan). Cells that were not treated with AAV or UTMD served as the control group.

### 4.9. Inhibition of Clathrin-Mediated Endocytosis

HeLa cells were cultured in 24-well plates and then incubated in DMEM containing CPZ (0.01 mg/mL, Sigma-Aldrich, St. Louis, MO, USA) for 1 h. Following 3 washes with PBS, the cells were incubated with AAV and treated with UTMD as described above. Control cells did not receive treatment with AAV or UTMD. After 1 h, cells were washed again with PBS and incubated in fresh DMEM.

### 4.10. Statistical Analysis

Data are expressed as mean ± SD. The independent samples t-test was used to detect differences between treated and control groups. *p* < 0.05 was considered statistically significant. Statistical analyses were performed using SPSS software (version 13.0, SPSS Inc., Chicago, IL, USA).

## 5. Conclusions

In the current study, UTMD enhanced gene transduction of AAV in a dose-dependent manner. UTMD facilitated uptake of viral particles into the cytoplasm and nucleus for long periods of time by stimulating endocytosis. UTMD stimulated the formation of clathrin CPs as well as nCPs. The mechanism of UTMD-stimulated endocytosis uncovered by our research shows that AAV does not enter the intracellular milieu by sonoporation exclusively and could further improve strategies for drug and gene delivery.
